# RNA-Seq Analysis of the Growth Hormone Transgenic Female Triploid Atlantic Salmon (*Salmo salar*) Hepatic Transcriptome Reveals Broad Temperature-Mediated Effects on Metabolism and Other Biological Processes

**DOI:** 10.3389/fgene.2022.852165

**Published:** 2022-05-23

**Authors:** Eric H. Ignatz, Tiago S. Hori, Surendra Kumar, Tillmann J. Benfey, Laura M. Braden, C. Dawn Runighan, Jillian D. Westcott, Matthew L. Rise

**Affiliations:** ^1^ Department of Ocean Sciences, Memorial University of Newfoundland and Labrador, St. John’s, NL, Canada; ^2^ Atlantic Aqua Farms Ltd., Charlottetown, PE, Canada; ^3^ Department of Biology, University of New Brunswick, Fredericton, NB, Canada; ^4^ AquaBounty Canada, Inc., Souris, PE, Canada; ^5^ Department of Pathology and Microbiology, Atlantic Veterinary College, University of Prince Edward Island, Charlottetown, PE, Canada; ^6^ Fisheries and Marine Institute, Memorial University of Newfoundland and Labrador, St. John’s, NL, Canada

**Keywords:** Atlantic salmon, metabolism, RNA-seq, transcriptomics, transgenic, triploid

## Abstract

This study examined the impact of rearing temperature (10.5, 13.5 or 16.5°C) on the hepatic transcriptome of AquAdvantage Salmon (growth hormone transgenic female triploid Atlantic salmon) at an average weight of 800 g. Six stranded PE libraries were Illumina-sequenced from each temperature group, resulting in an average of over 100 M raw reads per individual fish. RNA-sequencing (RNA-seq) results showed the greatest difference in the number of differentially expressed transcripts (1750 DETs), as revealed by both DESeq2 and edgeR (*q* < 0.05; fold-change > |1.5|), was between the 10.5 and 16.5°C temperature groups. In contrast, 172 and 52 DETs were found in the 10.5 vs. 13.5°C and the 13.5 vs. 16.5°C comparisons, respectively. Considering the DETs between the 10.5 and 16.5°C groups, 282 enriched gene ontology (GO) terms were identified (*q* < 0.05), including “response to stress”, “immune system process”, “lipid metabolic process”, “oxidation-reduction process”, and “cholesterol metabolic process”, suggesting elevated temperature elicited broad effects on multiple biological systems. Pathway analysis using ClueGO showed additional impacts on amino acid and lipid metabolism. There was a significant positive correlation between RNA-seq and real-time quantitative polymerase chain reaction (RT-qPCR) results for 8 of 9 metabolic-related transcripts tested. RT-qPCR results also correlated to changes in fillet tissue composition previously reported in these salmon (e.g., methionine and lysine concentrations positively correlated with *hsp90ab1* transcript expression), suggesting that rearing temperature played a significant role in mediating metabolic/biosynthetic pathways of AquAdvantage Salmon. Many transcripts related to lipid/fatty acid metabolism (e.g., *elovl2*, *fabpi*, *hacd2*, *mgll*, *s27a2*, *thrsp*) were downregulated at 16.5°C compared to both other temperature groups. Additionally, enrichment of stress-, apoptosis- and catabolism-relevant GO terms at 16.5°C suggests that this temperature may not be ideal for commercial production when using freshwater recirculating aquaculture systems (RAS). This study relates phenotypic responses to transcript-specific findings and therefore aids in the determination of an optimal rearing temperature for AquAdvantage Salmon. With approval to grow and sell AquAdvantage Salmon in the United States and Canada, the novel insights provided by this research can help industry expansion by promoting optimal physiological performance and health.

## Introduction

Global aquaculture production has grown 527% from 1990 to 2018 (FAO, 2020) and is now responsible for producing over half of the world’s seafood since the capture fisheries sector is unable to sustainably expand further to meet the increasing demand for food fish consumption (Cai and Leung, 2017; FAO, 2018). However, fish farming itself must be sustainable, and the industry must be proactive in developing new tools to maximize production and limit environmental impact.

Genetic modification is one option available that has sizeable potential to advance aquaculture and help meet the growing demand for safe, nutritious seafood. An example is the AquAdvantage Salmon (AAS), a growth hormone transgenic female triploid Atlantic salmon (*Salmo salar*). These reproductively sterile fish grow at approximately twice the rate of their non-transgenic siblings (Cook et al., 2000; Ganga et al., 2015). With lower food conversion ratios (FCRs), AAS also require less food to reach the same target weight (Tibbetts et al., 2013), further advancing their economic potential compared with conventional farmed Atlantic salmon. AquAdvantage Salmon are currently approved for production and sale within the United States and Canada, with regulatory approval obtained in 2015 and 2016, respectively.

Triploids may benefit the aquaculture industry, as they offer an effective, albeit not 100% guaranteed, option for reproductive sterility and genetic containment (Benfey, 2016). While conventional salmonids are pseudotetraploid due to incomplete restoration of diploidy following a whole-genome duplication event in the salmonid lineage (Lien et al., 2016), Atlantic salmon will be referred to as diploid, and where the second polar body is retained as triploid, to improve the paper’s readability. Growth performance between ploidies varies in salmonids, but there is a general trend that diploids grow faster during early development, while triploids catch up or surpass diploids in weight gain at harvest as they avoid the diversion of energy toward sexual development (Chiasson et al., 2009; Weber et al., 2014; Nuez-Ortín et al., 2017). However, it has been suggested that triploid salmonids have a lower optimum temperature for routine metabolism, feed intake and growth than diploids (Atkins and Benfey, 2008; Sambraus et al., 2017b; Sambraus et al.,2018), but other studies have found no such effect (Benfey et al., 1997; Bowden et al., 2018) or contradictory results (Ellis et al., 2013).

Further, recent results raised welfare concerns for the commercial production of conventional triploid Atlantic salmon, where reduced survival, higher levels of emaciation, and lower quality processing scores have been reported in Norway (Madaro et al., 2021). Therefore, it is of high importance for producers of triploid salmon to understand well how their fish will respond to varying environmental conditions during their production cycle. This information can then be used to develop production strategies that maximize growth potential while limiting stress and other negative consequences in recirculating aquaculture systems (RAS).

While recommendations have been made for the optimal rearing temperature of conventional diploid Atlantic salmon (Handeland et al., 2008; Hevrøy et al., 2013) and non-transgenic triploid Atlantic salmon (Sambraus et al., 2017a) in seawater, until recently, no information was available in this regard for AAS reared in freshwater RAS. It is now known what effect rearing temperature (i.e., 10.5, 13.5, and 16.5°C) has on the growth performance, nutrient utilization and innate antiviral response of AAS (Ignatz et al., 2020a; Ignatz et al., 2020b). Results showed that at 16.5°C, FCR was higher, dietary lipid deposition was diverted more to the viscera than to muscle, fillet yields were lower, and ω3 fatty acid deposition was less efficient, but weight gain was higher and faster than AAS reared at 10.5 and 13.5°C (Ignatz et al., 2020b). Further, when intraperitoneally injected with either polyriboinosinic polyribocytidylic acid [pIC; a synthetic double-stranded RNA (dsRNA) analog that elicits a potent antiviral-like response] or an equal volume of sterile phosphate-buffered saline (PBS), higher induction of target antiviral biomarker transcripts was generally found at 10.5°C than either 13.5 or 16.5°C (Ignatz et al., 2020a). Collectively, this suggests that rearing AAS at 16.5°C is not optimal for commercial production and that lower temperatures (i.e., 10.5 or 13.5°C) may be more suitable. However, until the current study, nothing was known about how temperature regulates the broader expression of liver transcripts in AAS.

The field of genomics offers powerful tools that can be used to assess different production models and help reveal the overall impact of changes in rearing conditions. In the past, microarrays have shown differences in hepatic gene expression between fast and slow-growing families of transgenic triploid Atlantic salmon (Xu et al., 2013). Multiple microarray platforms have also indicated changes in hepatic gene expression between transgenic coho salmon (*Oncorhynchus kisutch*) fed different ration levels compared to non-transgenic control fish (Rise et al., 2006). More recently, RNA-sequencing (RNA-seq) has emerged as a popular method allowing for the entire transcriptome to be assessed rather than a predefined number of transcripts (a potential limiting factor with microarrays). Several studies have examined the impact of elevated temperature on various tissue/organ transcriptomes in multiple fish species using RNA-seq (e.g., Tomalty et al., 2015; Tan et al., 2016; Veilleux et al., 2018; Shi et al., 2019; Bowen et al., 2020; Pan et al., 2021; Quan et al., 2021; Yang et al., 2021; Zhou et al., 2021). However, most of these studies measured responses following acute exposure (i.e., hours to days) to thermal extremes and there is limited information on transcriptomic signatures of fish reared long-term (i.e., months) within typical thermal ranges observed in aquaculture.

As part of the objective to determine the optimal rearing temperature for the commercial production of AAS, RNA-seq was used to compare the hepatic transcriptomes of AAS reared at either 10.5, 13.5 or 16.5°C at an average weight of 800 g. This is the first transcriptomic study to examine the impact of temperature on this transgenic line of salmon and provides novel insight into the mechanisms behind the phenotypic differences previously described in these fish. Not only does this research elucidate hepatic gene expression responses observed in AAS, but the results generated shed new light on the molecular pathways involved in how salmonids respond to differences in thermal conditions.

## Materials and Methods

### Experimental Animals and Rearing Conditions

A full description of the production and rearing conditions of fish used in the current study can be found in Ignatz et al. (2020b). Briefly, all fish were from St. John River stock and were hatched and reared at AquaBounty Canada (PE, Canada). AAS were produced from a specific transgenic line (termed EO-1α) containing a single copy of a gene construct (opAFP-GHc2) consisting of an ocean pout (*Macrozoarces americanus*) antifreeze protein promoter and the coding sequence for the Chinook salmon (*Oncorhynchus tshawytscha*) growth hormone gene (Yaskowiak et al., 2007, Yaskowiak et al., 2006). A single homozygous transgenic sex-reversed neomale (i.e., functionally masculinized genetic female) was crossed with three non-transgenic females, producing all-female transgenic offspring. The neomale parent was originally produced through oral delivery of 17α-methyltestosterone for a limited period during juvenile rearing. All fertilized eggs were pooled together before subjection to hydrostatic pressure shock to induce triploidy, making the fish reproductively sterile (AquaBounty Technologies, Inc., 2010; Benfey, 2016).

AAS were grown from first feeding fry (starting weight of ∼0.51 g) in 1.5 m^3^ tanks at 10.5°C, 13.5°C, and 16.5°C (±0.5°C) in triplicate. De-gassed and oxygenated freshwater from a well (<1 g/L salinity) was supplied to each tank in a RAS (10%–20% make-up daily), and dissolved oxygen was measured daily and maintained at >9.0 mg/L. Fish were fed commercial diets throughout the day via automatic feeders with continuous light provided. Passive integrated transponder (PIT) tags (Avid Canada Corp., Calgary, AB, Canada) were previously inserted into the peritoneal cavities of each fish when they averaged 400 g. At an average weight of 800 g, liver samples were collected to determine the overall impact of temperature on the hepatic transcriptome of AAS. Samplings took place approximately 10, 11, and 12 months after the onset of the experiment in the 16.5, 13.5, and 10.5°C treatments, respectively.

### Sample Collection

Fish were taken off feed at least 24 h in advance of sampling, and fish were euthanized by an overdose (0.4 g/L) of anesthetic (MS-222; Syndel Canada, Nanaimo, BC, Canada), buffered with an equal mass of sodium bicarbonate. As salmon at each rearing temperature reached an average weight of 800 g, fish were sampled by convenience, in that the first salmon found that fell within 700–900 g were sampled. Liver samples, taken from the most distal portion of the posterior lobe, were collected using standard aseptic techniques. Samples were placed in 1 ml of RNA*later*™ (Sigma-Aldrich, Oakville, ON, Canada) kept on ice, then stored at 4°C overnight. The RNA*later* was subsequently removed, and samples were stored at −70°C until RNA extraction. Twenty-one liver samples were collected per rearing temperature, but only two samples per tank (*n* = 6 per temperature treatment) were chosen for subsequent analyses. The sample list was narrowed down to only fish that fit within ±1 standard deviation from the mean weight calculated after sampling and, if more than two fish per tank remained, then two were randomly chosen.

Fillet samples were collected concurrently during each sampling period from the same fish sampled for liver transcript expression. Fillets were maintained on ice in plastic bags and subsequently stored at −70°C until samples were shipped for compositional analysis. A full description of how fillet composition was analyzed and the results of those analyses can be found in Ignatz et al. (2020b).

### RNA Extraction and Purification

RNA extractions were performed using the RNeasy® Mini Kit (Qiagen, Mississauga, ON, Canada), and the TURBO DNA-free™ Kit (Invitrogen, Burlington, ON, Canada) was utilized for DNase digestion in solution. All procedures were completed according to the manufacturers’ instructions, with small adjustments made based on guidelines found in the Qiagen Purification of Total RNA Using the RNeasy® Fibrous Tissue Mini Kit Protocol. RNA quantity and quality were first assessed using spectrophotometry and 0.7% agarose gel electrophoresis, respectively. All samples showed evidence of high-quality RNA (A260/280 & A260/230 ratios > 2.0, with distinct 18S/28S ribosomal RNA bands) before shipment to the Centre d’expertise et de services Génome Québec (Montréal, QC, Canada) for further analysis. RNA Integrity Numbers (RIN), assessed using a Bioanalyzer 2100 (Agilent, Santa Clara, CA, United States), of all RNAs used for sequencing and qPCR were found to be ≥8.9.

### Sequencing, Read Alignment and Annotation

Library construction and sequencing were performed at Génome Québec CES, with bioinformatics support provided by the Canadian Centre for Computational Genomics (C3G; Montréal, QC, Canada) using their GenPipes next-generation sequencing data processing framework (Bourgey et al., 2019). A total of 18 stranded PE100 libraries were created from the six RNA samples per rearing temperature (Table 1) using the NEBNext Ultra kit. RNA sequencing was completed using three lanes (six libraries per lane) of a flow cell in an Illumina® HiSeq4000 sequencing platform. Trimming of the resulting reads were performed using Trimmomatic (v.0.36) (Bolger et al., 2014). Reads were trimmed from the 3′ end with a Phred score cut-off of 30. Illumina sequencing adapters were removed from the reads, and all reads were required to have a length of at least 32 bp. The filtered reads were then aligned to a reference genome (*Salmo salar* assembly ICSASG_v2 taken from NCBI) using STAR (v2.5.3a) (Dobin et al., 2013), creating Binary Alignment Map (BAM) files. All BAM files from the same sample were merged into one single BAM file using Picard (v.2.9.0) (Picard Tools, 2019). Wiggle tracks format (wig) files were generated from the merged file using bedGraphToBigWig (ucsc-userApps v.346) (Kent et al., 2010). Cufflinks (v.2.2.1) (Roberts et al., 2011) was used to assemble aligned RNA-Seq reads into transcripts and estimate their abundance as fragments per kilobase of exon per million fragments mapped (FPKM). Individual count data were extracted by multiplying each Cuffnorm sample count table by the library size factors to de-normalize the counts using a method as in Myers et al. (2021). Once transcripts were assembled and their corresponding FPKM estimated, they were annotated with a known reference set of transcripts obtained from the Ensembl database (Accession GCA_000233375). The transcript list was also annotated with putative *Homo sapiens* ortholog RefSeq mRNA accession identifiers based on lowest BLASTx score with an Expect (E) value cut-off <1e-5 to better assist functional annotation, which was able to successfully annotate ∼79% of the assembled transcriptome. Typically, non-model species’ gene ontology (GO) annotations are assigned by orthology and lack experimental evidence. Human annotations were added in this study as they are more reliable and allowed for further downstream analyses to be conducted (i.e., GOrilla, ClueGO).

**TABLE 1:** Trimming and alignment metrics of each biological replicate RNA-seq library.

	Library name	SRA^a^ accession	Raw reads #	Surviving reads # (%)	Aligned reads (%)	Alternative alignments (%)	% Coverage	% Exonic rate
16.5°C	S1_16.5	SRR16105563	112,257,950	112,227,600 (99.97)	108,739,280 (96.89)	43,960,352 (40.43)	30.40	86.26
	S2_16.5	SRR16115861	114,484,598	114,440,190 (99.96)	110,338,704 (96.42)	35,997,993 (32.63)	32.03	85.43
	S3_16.5	SRR16122595	101,880,276	101,848,592 (99.97)	97,969,194 (96.19)	30,641,463 (31.28)	28.69	85.52
	S4_16.5	SRR16958631	107,258,770	107,234,866 (99.98)	103,748,543 (96.75)	29,735,650 (28.66)	30.54	86.03
	S5_16.5	SRR16958615	117,304,540	117,277,318 (99.98)	113,299,533 (96.61)	39,245,504 (34.64)	32.36	86.36
	S6_16.5	SRR16975862	98,692,524	98,668,078 (99.98)	95,693,153 (96.99)	31,917,863 (33.35)	27.78	86.34
13.5°C	S1_13.5	SRR16988317	105,027,464	105,000,658 (99.97)	101,581,042 (96.74)	33,599,049 (33.08)	28.46	86.15
	S2_13.5	SRR17038623	74,841,184	74,821,020 (99.97)	72,261,894 (96.58)	20,308,874 (28.11)	20.51	85.17
	S3_13.5	SRR17481977	109,834,502	109,803,350 (99.97)	106,465,002 (96.96)	32,170,009 (30.22)	30.64	86.62
	S4_13.5	SRR17503995	90,177,906	90,155,004 (99.98)	87,224,875 (96.75)	29,904,438 (34.28)	24.55	86.53
	S5_13.5	SRR17481976	115,909,552	115,859,460 (99.96)	111,534,501 (96.27)	35,826,986 (32.12)	30.88	86.18
	S6_13.5	SRR17500070	105,921,688	105,894,066 (99.97)	102,319,865 (96.63)	32,417,461 (31.68)	28.55	85.75
10.5°C	S1_10.5	SRR16097286	99,113,996	99,077,664 (99.96)	95,533,239 (96.42)	24,867,589 (26.03)	28.39	86.84
	S2_10.5	SRR17446820	92,641,680	92,565,332 (99.92)	88,928,239 (96.07)	25,834,830 (29.05)	26.18	85.41
	S3_10.5	SRR17500116	94,647,236	94,620,554 (99.97)	91,837,075 (97.06)	24,089,272 (26.23)	26.81	87.02
	S4_10.5	SRR17469864	100,106,994	100,071,212 (99.96)	96,974,288 (96.91)	26,257,994 (27.08)	28.24	86.65
	S5_10.5	SRR17504015	93,838,306	93,807,530 (99.97)	90,983,847 (96.99)	26,985,379 (29.66)	26.65	86.46
	S6_10.5	SRR17500112	106,725,990	106,685,144 (99.96)	102,643,077 (96.21)	28,060,332 (27.34)	30.58	87.37

a Sequence read archive.

### Identification of Differentially Expressed Transcripts and GO Term Enrichment Analysis

Utilizing the transformed read counts obtained from Cuffnorm, differentially expressed transcripts (DETs) were found using the online Galaxy (Afgan et al., 2018) platform. DESeq2 (Love et al., 2014) under Galaxy Version 2.11.40.2 was used with outlier and independent filtering to test pairwise comparisons of rearing temperature first. Then, edgeR (Robinson et al., 2009) under Galaxy Version 3.20.7.2, without filtering lowly expressed genes, was used to perform the exact same comparisons. As count data exist as compositions, this presents a challenge to normalizing results and comparing differential expression (Quinn et al., 2018). DESeq2 and edgeR analyses were compared to obtain higher confidence as they use the same reasonable assumption that most genes will not be differentially expressed between treatments but apply different approaches to estimate dispersion (Hall et al., 2021). By using both programs, more robust observations can be made between comparisons. Once each DET list was obtained from both DESeq2 and edgeR, they were filtered by their false discovery rate (FDR) (*q*-value) to keep only transcripts with values less than 0.05, as well as fold-change values above |1.5|. The matched pairwise comparisons by DESeq2 and edgeR were then compared, and only filtered DETs found by both programs were ultimately used for GO term enrichment analysis (GTEA). Comparisons between different gene lists were accomplished through VENNY (v.2.1) (Oliveros, 2007).

With the transcriptome annotated with *Homo sapiens* putative orthologs, the concordant lists of DETs found by DESeq2 and edgeR were used as input for the GOrilla platform (Eden et al., 2009; Eden et al., 2007), with both target and background lists present in the analysis. Only GO terms with *q*-values below 0.05 were considered significant. Pathway analysis was performed using the ClueGO (v.2.5.7) (Bindea et al., 2009) Cytoscape (v.3.7.2) plugin, illustrating enriched GO (Biological Process, Molecular Function, Cellular Component; v.08.05.2020) and Reactome pathway (v.08.05.2020) terms among annotated DETs. Analyses were performed using a two-sided hypergeometric test after a Bonferroni step-down *p*-value adjustment. Networks were also designed using a kappa statistic threshold of 0.4 and a medium specificity level, and only including network terms with *p* < 0.05.

### RT-qPCR Validation

Nine transcripts were chosen for RT-qPCR validation of RNA-seq results based on their GO terms. Selected GO terms were lipid/fatty acid metabolism (*elovl2*, *fabpi*, *hacd2*, *mgll*, *s27a2*, *thrsp*), protein metabolism (*hgfa*, *hsp90ab1*) and oxidoreductase activity (*gstk1*). These areas of focus were selected based on past interest in nutrient utilization (Tibbetts et al., 2013; Ganga et al., 2015; Ignatz et al., 2020b) and oxidation reduction processes of growth hormone transgenic Atlantic salmon (Xu et al., 2013). All transcripts chosen for validation were also found significantly differentially expressed (*q* < 0.01) by both DESeq2 and edgeR, with fold-changes between the 10.5°C and 16.5°C treatments > |2.0|. One target gene primer pair, and all normalizer transcript primers, were designed from previous studies (Jones et al., 2007; Morais et al., 2009; Xue et al., 2015; Caballero-Solares et al., 2017), while all remaining target transcript primers were designed using NCBI’s Primer-Blast (Ye et al., 2012). cDNA was synthesized from 1 μg of purified RNA (the same extracted RNA that was sent for sequencing) using the iScript™ cDNA synthesis kit (Bio-Rad; Saint-Laurent, QC, Canada) following the manufacturer’s instructions. To assess potential gDNA contamination, no reverse transcriptase (no-RT) controls were also performed using pooled RNA of randomized samples (*n* = 9 per pool). Every primer set was quality-tested using a cDNA pool derived from equal quantity input from each sample to determine amplification efficiencies (Pfaffl, 2001) with a 5-fold, 6-point dilution series in triplicate. Gel electrophoresis also confirmed single products and amplicon size (74–192 bp) for each primer pair. A single peak was found in each dissociation curve with no evidence of primer dimers, no gDNA contamination was found in no-RT controls, and amplification efficiencies ranged between 92 and 110%. All information related to the primers that were utilized in this study is shown in Table 2. Based on amplification results from QC testing, cDNA was diluted in nuclease-free water (Invitrogen) at 1:5 or 1:25 concentrations before RT-qPCR analysis for lower and higher expressed transcripts, respectively.

**TABLE 2:** RT-qPCR primers.

Gene name (Symbol) (GenBank accession number)	Nucleotide sequence (5′-3′)	Efficiency^a^ (%)	*r* ^2^	Amplicon size (bp)	Source
Elongation of very long chain fatty acids protein 2 (*elovl2*) (TC91192)^b^	F: CGG​GTA​CAA​AAT​GTG​CTG​GT	100.9	0.983	145	Morais et al. (2009)
	R: TCT​GTT​TGC​CGA​TAG​CCA​TT				
Fatty acid-binding protein, intestinal-like (*fabpi*) (XM_014206454)	F: CCT​GGG​CGT​ACA​GTT​TGA​CT	100.9	0.998	164	This study
	R: TAT​AGC​TCT​GTA​CTA​GCT​CTC​CTC​C				
Glutathione S-transferase kappa 1 (*gstk1*) (XM_014154124)	F: GGA​GTG​GAC​ATC​AGC​ATC​AGT	92.8	0.960	197	This study
	R: ACA​CCT​TAT​GAT​GCC​AGA​GGA​A				
3-hydroxyacyl-CoA dehydratase 2 (*hacd2*) (XM_014165367)	F: CAG​ACC​GGA​GCT​CTT​CTG​G	104.1	0.975	145	This study
	R: TGT​CTT​CAT​TCT​GGA​CCT​CTC​G				
Hepatocyte growth factor a (*hgfa*) (NM_001140139)	F: CAG​ACG​GGG​ACA​AGA​TGC​C	110.2	0.999	104	This study
	R: CCG​CGA​AGA​AGA​TAA​ACG​CA				
Heat shock protein 90 alpha family class B member 1 (*hsp90ab1*) (NM_001123532)	F: AGC​CTC​ACG​TTT​TTC​CAA​TCG	92.4	0.978	150	This study
	R: TGC​GTT​GCC​CAC​CAT​TAA​CT				
Monoglyceride lipase (*mgll*) (NM_001140001)	F: CTT​GTC​AGT​AAT​CTT​TGG​ACC​CCT​A	107.7	0.997	192	This study
	R: ATG​CCT​CTG​TGA​ATT​GCG​CT				
Very long-chain acyl-CoA synthetase (*s27a2*, alias *slc27a2*) (NM_001141797)	F: TTC​ACC​CAG​AAG​CAT​AGG​AGC	100.3	0.999	75	This study
	R: GGC​CAA​ACT​GGC​AAA​AAG​GA				
Thyroid hormone responsive (*thrsp*) (XM_014163692)	F: CTA​CCG​GGA​ACA​GCC​AGA​AA	106.9	0.995	74	This study
	R: GCA​TAG​TGT​TGG​ACT​CGG​CA				
Elongation factor 1 alpha (*ef1a*) (NM_001141909)^c^	F: GTG​GAG​ACT​GGA​ACC​CTG​AA	97.4	0.997	155	Jones et al. (2007)
	R: CTT​GAC​GGA​CAC​GTT​CTT​GA				
Polyadenylate-binding protein 1 (*pabpc1*) (EG908498)^c^	F: TGA​CCG​TCT​CGG​GTT​TTT​AG	99.2	0.999	108	Caballero-Solares et al. (2017)
	R: CCA​AGG​TGG​ATG​AAG​CTG​TT				
60S ribosomal protein 32 (*rpl32*) (BT043656)^d^	F: AGG​CGG​TTT​AAG​GGT​CAG​AT	99.6	0.995	119	Xue et al. (2015)
	R: TCG​AGC​TCC​TTG​ATG​TTG​TG				

a Amplification efficiencies were calculated using a 6-point 1:5 dilution series starting with an equal mixture of cDNA from every sample.

b Atlantic salmon Gene Index identification.

c Normalizer genes chosen for this study.

d Normalizer genes tested, but ultimately not chosen for this study.

qPCR amplification was performed using a SsoAdvanced™ Universal SYBR® Green Supermix qPCR kit (Bio-Rad) per the manufacturer’s instructions with a CFX-384 thermal cycler (Bio-Rad). The master mix was loaded using an Aurora automated plate dispenser with VERSAwar 10.v.1.2.48 software, and samples were plated in triplicate using an electronic Sartorius Picus® pipette. The total reaction volume was 11 μl, comprised of: 5 μl SYBR® Green Supermix, 4 μl nuclease-free water, 0.5 μl forward primer (10 μM), 0.5 μl reverse primer (10 μM), and 1 μl cDNA template (2 or 10 ng input RNA, depending on dilution concentration). PCR amplification and cycling were performed using the same programs described in Ignatz et al. (2020a). Each gene was run on a single plate, which included no-template controls (NTC).

Raw expression profiles were imported into qbase+ (Biogazelle; Gent, Belgium) (Hellemans et al., 2007), and technical replicates falling outside of ±0.5 cycle threshold (CT) from two close replicates were removed. A total of three normalizer genes were tested (*ef1a*, *pabpc1*, *rpl32*); however, only *ef1a* and *pabpc1* were chosen as the final study normalizers as that combination showed the highest stability (geNorm M value and coefficient of variation of 0.438 and 0.154, respectively) (Vandesompele et al., 2002). Normalized relative quantities (NRQs) (Vandesompele et al., 2002) were calculated using amplification efficiencies for each primer pair (Table 2), which were then log_2_ transformed in Microsoft Excel.

### Statistical Procedures

Statistical comparison of treatment means was performed by one-way ANOVA, testing the influence of temperature on NRQs. Tukey’s post-hoc tests were performed where significant differences were detected. Linear regression, conducted in Excel, was used to model the relationship between fold-change values calculated from RNA-sequencing and those from the RT-qPCR validation study. Generation of hierarchical clustering, correlogram and principal component analysis (PCA) diagrams, together with ANOVA testing, were performed in R (version 3.6.3) (R Studio Team, 2015). Statistical differences were considered significant at *p* < 0.05.

Hierarchical clustering heatmaps applied standardized FPKM values, calculated using the following equation:

$${\text{z}}_{\text{i}}=\frac{x_{i}-\text{min}\left(x\right)}{\max\left(x\right)-\text{min}\left(x\right)}$$

where *x*_*i*_ is a specific FPKM value for a given sample and *x* is the range of FPKM values across samples for a given transcript used to calculate an individual standardized value (z_i_).

## Results

### Differentially Expressed Transcripts

**FIGURE 1:**
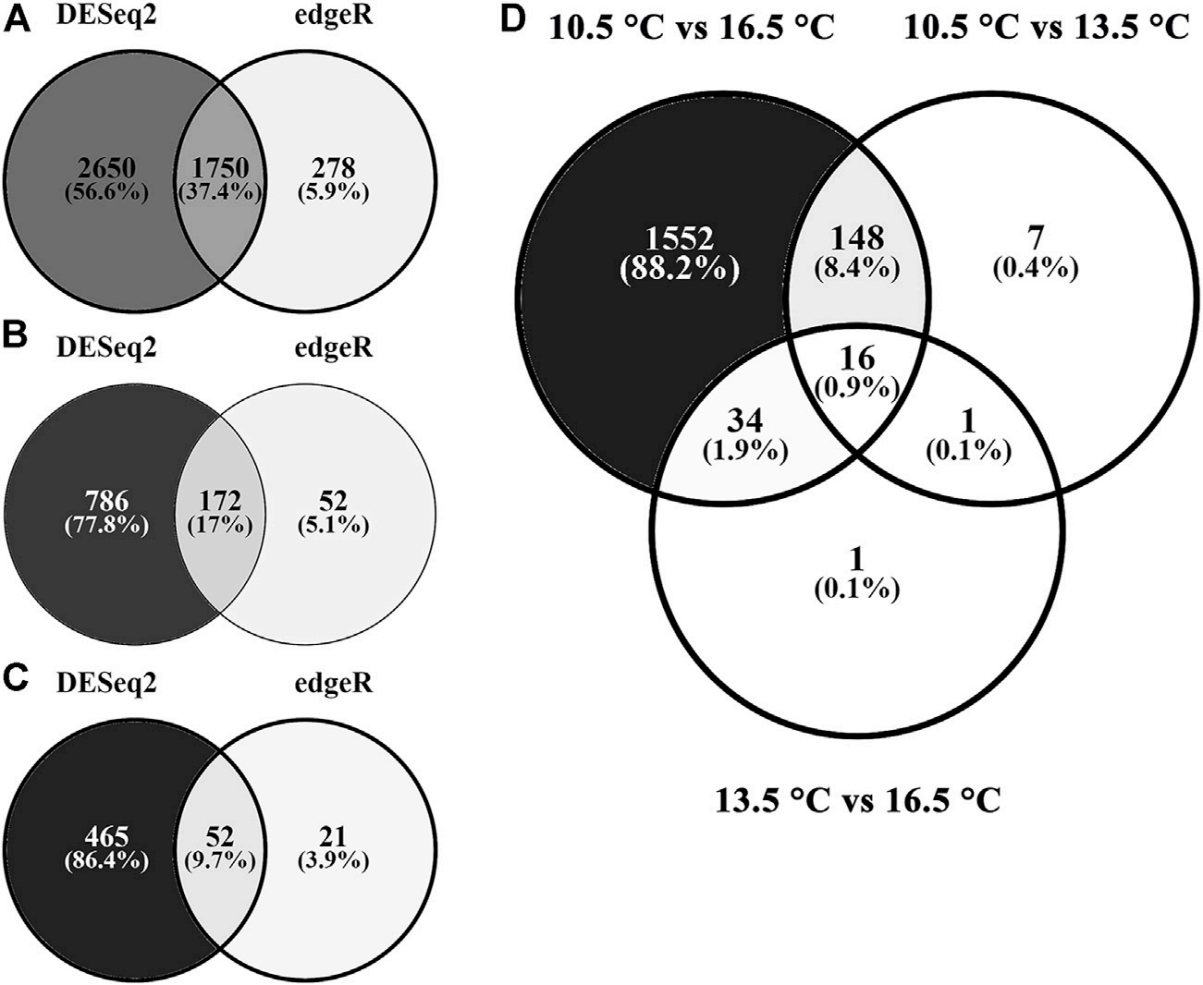
Differential transcript expression in response to rearing temperature. Venn diagrams depict the number of shared and separate differentially expressed (both up- and down-regulated; FDR-adjusted *p* < 0.05) transcripts identified by either DESeq2 (left), edgeR (right), or those in common (middle). Temperature comparisons are presented as **(A)** 10.5°C vs. 16.5°C, **(B)** 10.5°C vs. 13.5°C, **(C)** 13.5°C vs. 16.5°C, and **(D)** differentially expressed transcripts shared between both DESeq2 and edgeR for each pairwise comparison.

On average, more than 100 M raw reads per sample were generated from sequencing (Table 1). For all samples, >96% of those reads survived the trimming process and were aligned to the reference *Salmo salar* genome. The final read counts were then pairwise compared between each rearing temperature treatment (Figures 1A–C) using both DESeq2 and edgeR. In all comparisons, DESeq2 found more DETs than the edgeR program (both analyzed with significance determined at *q*-value < 0.05, fold change ≥ |1.50|). Full DET lists with DESeq2, edgeR and concordant results can be found in Supplementary Table S1 (10.5 vs. 16.5°C), Supplementary Table S2 (10.5 vs. 13.5°C) and Supplementary Table S3 (13.5 vs. 16.5°C). Only concordant DET lists for each comparison were used in subsequent analyses. Overlap between pairwise lists is shown in Figure 1D. Overall, the highest number of DETs was found in the comparison between the 10.5 and 16.5°C treatments (1750 DETs), followed by the 10.5 vs. 13.5°C (172 DETs) and the 13.5 vs. 16.5°C (52 DETs) assessments. Only 16 DETs were found in all three pairwise tests (i.e., found significantly different among all three temperature groups).

**FIGURE 2:**
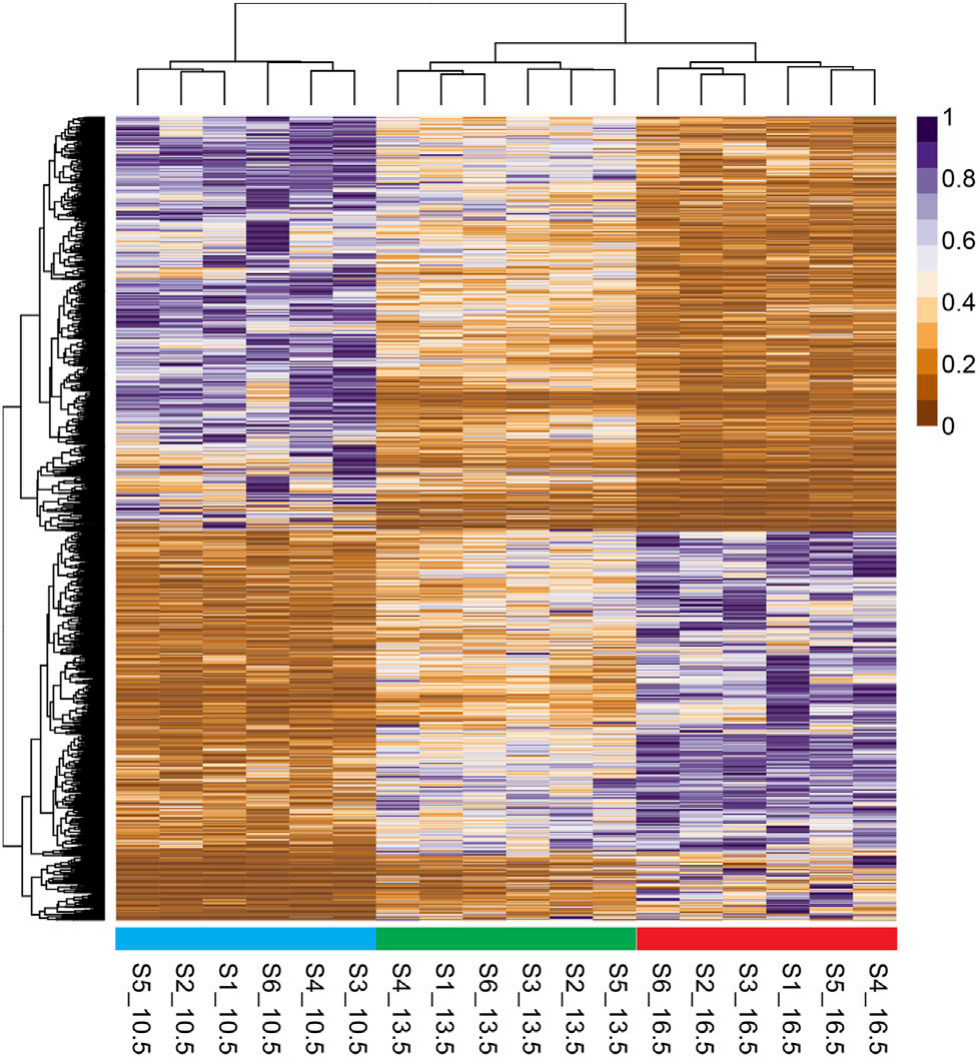
Heatmap and hierarchical clustering of differentially expressed transcripts (1759 DETs) found in comparison of all three rearing temperature treatments. RNA samples used in sequencing are labeled on the base of the figure, with standardized fragments per kilobase of transcript per million mapped reads (FPKM) values for each DET clustered along the vertical axis.

A heatmap with hierarchical clustering of standardized FPKM values of all 1759 DETs found in this study is shown in Figure 2. All three temperature groups clustered separately from one another; however, the 13.5 and 16.5°C samples share the same initial branching point.

### Gene Ontology Term Enrichment Analysis

**FIGURE 3:**
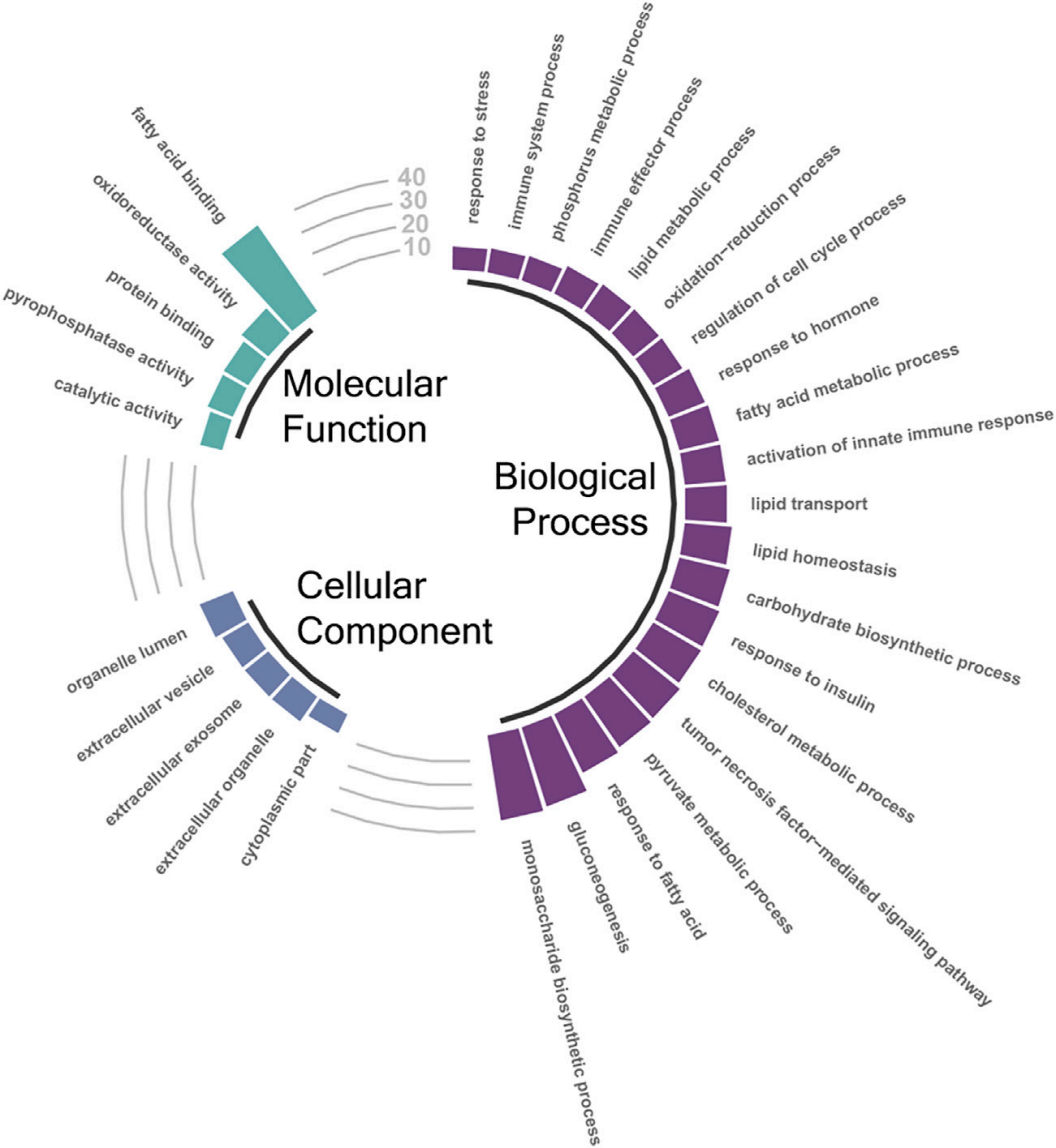
Selected significantly enriched (FDR-adjusted *p* < 0.05) gene ontology (GO) terms associated with significantly differentially expressed transcripts (DETs; both up- and down-regulated) between the 10.5°C and 16.5°C groups. The height of the bars represents the percentage of DETs found in the current study compared to total number of transcripts assigned to that specific GO term. A full list of enriched GO terms identified between the 10.5°C and 16.5°C groups can be found in Supplementary Table S4.

Taking the DETs that were found significant by both DESeq2 and edgeR, GTEA was performed on each pairwise list. The full results from each of those comparisons can be found in Supplementary Table S4 (10.5 vs. 16.5°C), Supplementary Table S5 (10.5 vs. 13.5°C), and Supplementary Table S6 (13.5 vs. 16.5°C). Due to the 10.5 vs. 16.5°C comparison containing the highest number of DETs, we focused on these results. A total of 225 Biological Process, 24 Molecular Function, and 33 Cellular Component GO terms were enriched in the annotated DET list between the 10.5 and 16.5°C groups. Figure 3 illustrates selected significantly enriched (*q*-value < 0.05) GO terms from this specific assessment. Notably, GO terms such as “response to stress”, “immune system process”, “lipid metabolic process”, “oxidation-reduction process”, and “cholesterol metabolic process” were all enriched, suggesting that elevated temperature elicited broad effects on multiple biological systems in the liver tissues of AquAdvantage Salmon.

### Pathway Analysis

**FIGURE 4:**
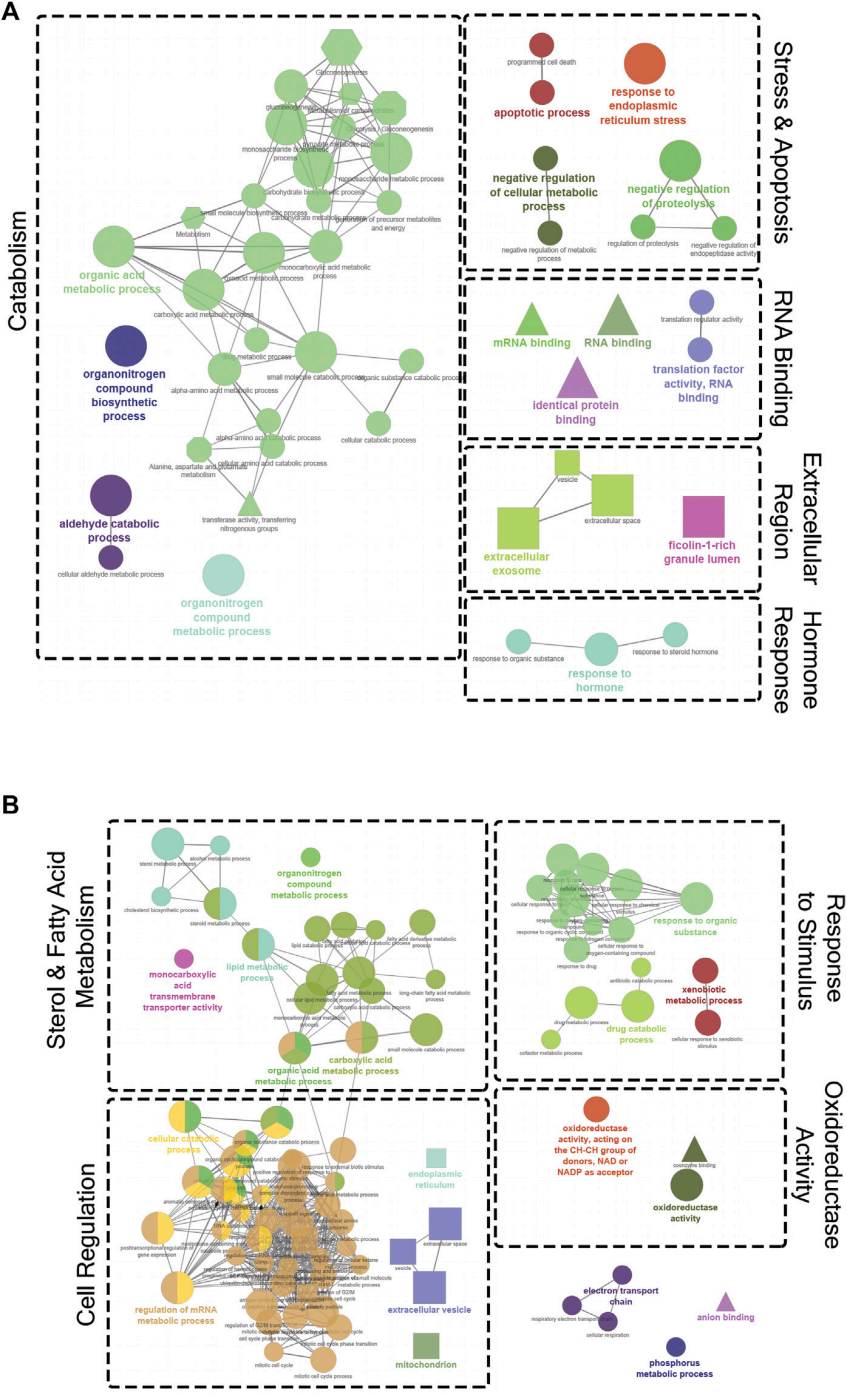
Gene ontology (GO) term enrichment and pathway network analysis of significantly differentially expressed transcripts (DETs) between the 10.5°C and 16.5°C groups. **(A)** Downregulated DETs at 10.5°C (659 DETs). **(B)** Upregulated DETs at 10.5°C (718 DETs). Only significant (*p* < 0.05) network terms were included in the visualization. The diameter of each node indicates the level of significance for that specific term, with a larger node corresponding to a lower *p*-value. Node colour signifies related processes that share similar DETs. The shape of the node reflects the database where the term originated (circle, GO Biological Process; triangle, GO Molecular Function; square, GO Cellular Component; hexagon, Reactome). Dotted borders and labels were used to group related clusters and highlight general themes.

Figure 4 shows the results of pathway analyses conducted using both GO and Reactome terms from the annotated DET list comparing the 10.5 and 16.5°C treatments. In Figure 4A, all the enriched terms downregulated at 10.5°C (i.e., upregulated at 16.5°C) are shown. These terms were grouped into five general categories: catabolism, stress and apoptosis, RNA binding, extracellular region, and hormone response. In contrast, Figure 4B shows all the upregulated terms at 10.5°C (i.e., downregulated at 16.5°C). These terms mostly fit into four general categories: sterol and fatty acid metabolism, cell regulation, response to stimulus, and oxidoreductase activity.

**FIGURE 5:**
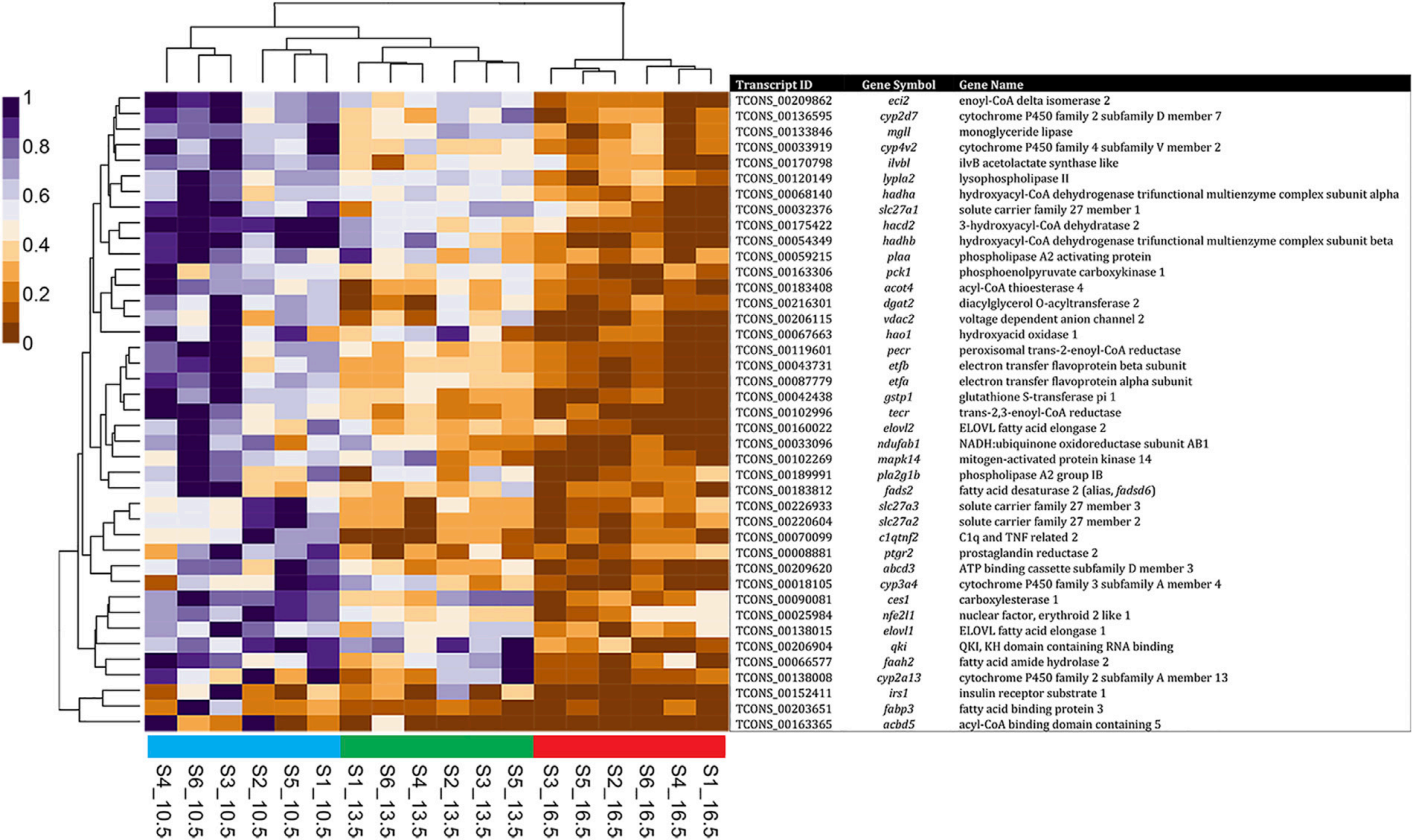
Heatmap and hierarchical clustering of non-redundant differentially expressed transcripts (41 DETs) found in comparison of the 10.5°C and 16.5°C groups associated with GO: 0006631—Fatty Acid Metabolic Process. RNA samples used in sequencing are labeled on the base of the figure, with standardized fragments per kilobase of transcript per million mapped reads (FPKM) values for each DET clustered along the vertical axis.

Examining the transcripts associated with the GO term “fatty acid metabolic process” found during this analysis, Figure 5 shows that many of these transcripts were upregulated at 10.5°C when compared to 16.5°C. Furthermore, fish reared at 13.5°C had an intermediate response for these transcripts, but overall, their clustering showed a closer relationship to the 10.5°C group than 16.5°C. Further, the relationships between GO terms “apoptotic process” (Supplementary Figure S1) and “oxidoreductase activity” (Supplementary Figure S2) revealed that at elevated temperature (i.e., 16.5°C), salmon expressed more transcripts related to apoptosis and less transcripts associated with oxygen reduction processes compared to the lower two temperature groups (particularly 10.5°C).

### RT-qPCR Validation

**FIGURE 6:**
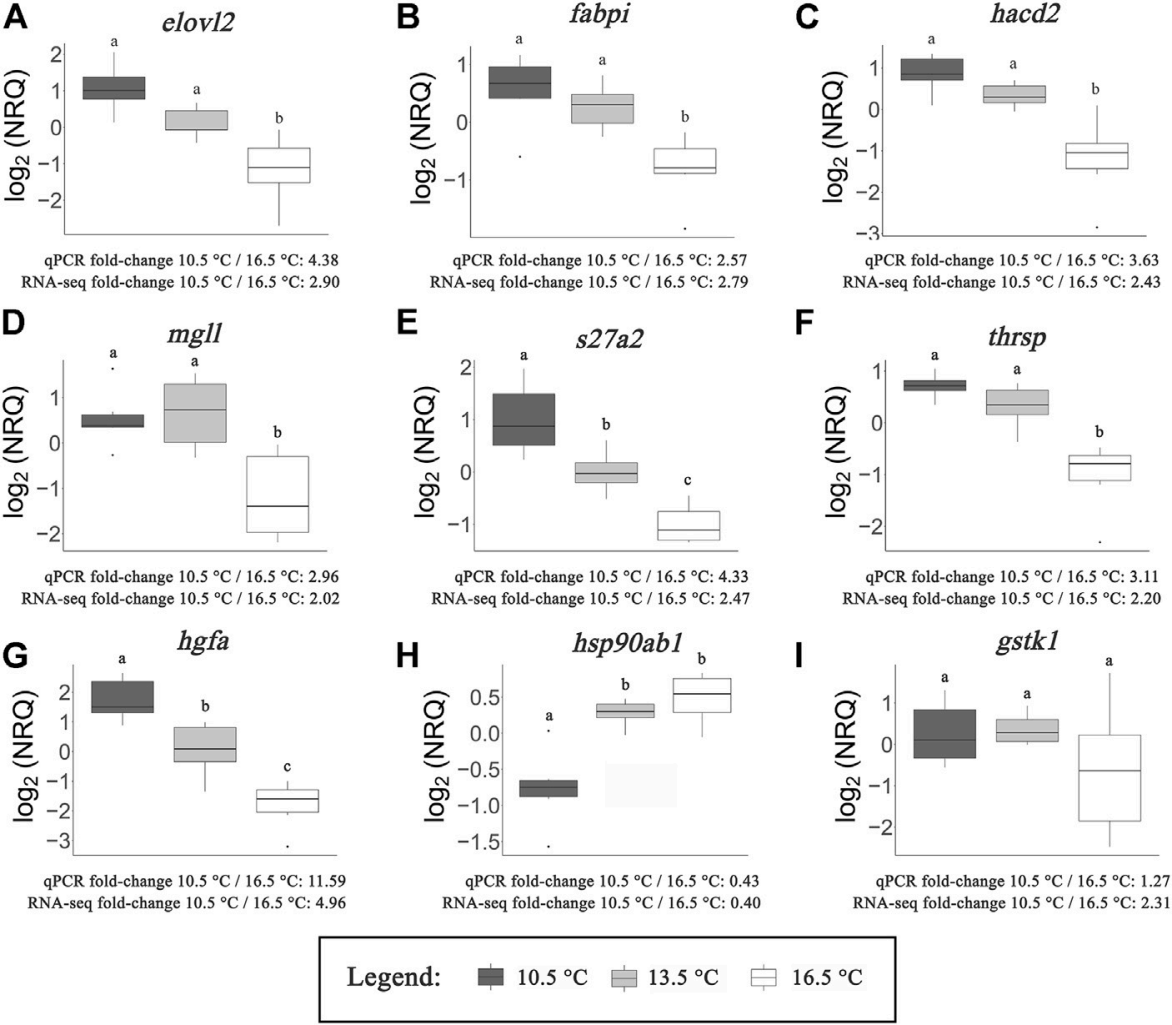
qPCR results of targeted transcripts involved in fatty acid/lipid metabolism **(A–F)**, protein metabolism **(G,H)** and oxidoreductase activity **(I)**. Normalized relative quantities (NRQs) are provided log_2_ transformed. Letters represent significant differences (*p* < 0.05) between rearing temperatures as determined by one-way ANOVA and subsequent Tukey’s post-hoc tests. Average fold-change values calculated from qPCR results and RNA-seq data (average between DESeq2 and edgeR) are provided in comparison of the 10.5°C and 16.5°C treatments.

**FIGURE 7:**
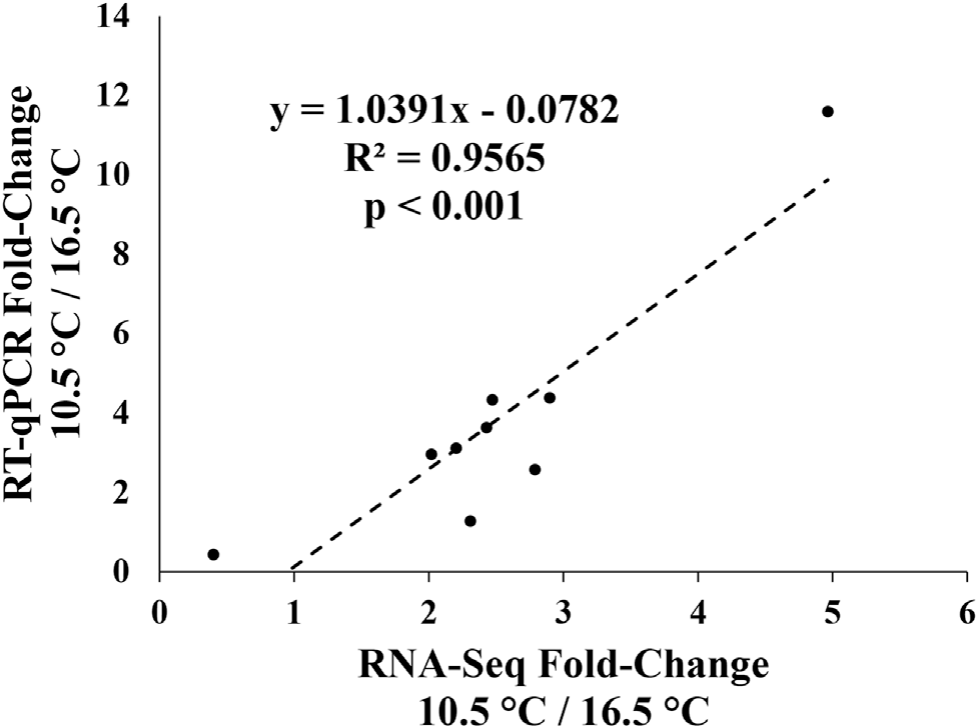
Regression plot of transcript expression fold-changes between the 10.5°C and 16.5°C treatments calculated from the RNA-seq dataset (average between DESeq2 and edgeR) and normalized relative quantity (NRQ) values from the qPCR-validation.

Figures 6A–I show the expression of nine metabolic-related transcripts chosen for RT-qPCR validation. All but one of the transcripts demonstrated significantly correlated fold-change differences between the 10.5 and 16.5°C temperature groups between RT-qPCR and RNA-seq results, with *gstk1* being the one exception. This result is further visualized in Figure 7, where an overall highly significant (*p* < 0.001) positive correlation (*R* ^2^ = 0.96) between both methods measuring transcript expression was found. Therefore, we concluded that the RNA-seq results of this study were successfully validated.

All transcripts associated with fatty acid/lipid metabolism (Figures 6A–F) demonstrated significant upregulation at 10.5 and 13.5°C compared to 16.5°C. In five of these six cases (*elovl2*, *fabpi*, *hacd2*, *mgll*, *thrsp*), transcript expression did not significantly differ between the 10.5 and 13.5°C temperature groups. However, the expression of *s27a2* showed significant differences between each treatment, indicating an inverse relationship with temperature. Although lacking statistical significance, this trend was also evident in four of the other transcripts (*elovl2*, *fabpi*, *hacd2*, *thrsp*). For the two remaining transcripts that successfully validated that are related to protein metabolism (Figures 6G,H), opposing patterns emerged. Expression of *hgfa* exhibited an inverse relationship to rearing temperature, with significant differences between all three treatments. In contrast, *hsp90ab1* was expressed more highly at both 13.5 and 16.5°C compared to 10.5°C. While the one transcript related to oxidoreductase activity (*gstk1*; Figure 6I) failed to successfully validate, it was still clear that several fish reared at 16.5°C exhibited lower expression of this transcript compared to the other two temperature groups.

### Multivariate Analyses

**FIGURE 8:**
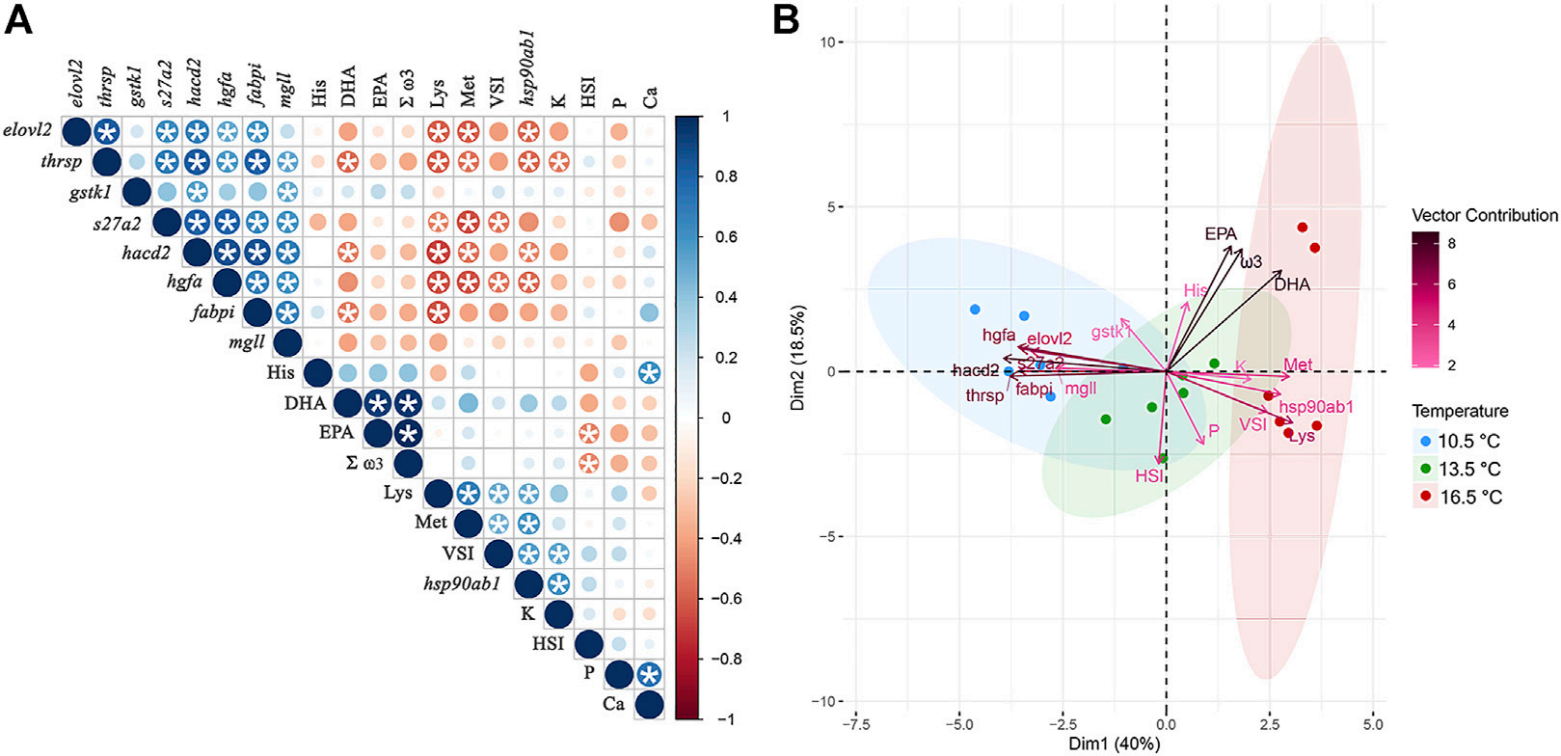
**(A)** Correlogram of expression profiles for all transcripts used for RT-qPCR validation alongside fillet fatty acid (ω3, total omega-3; DHA, docosahexaenoic acid; EPA, eicosapentaenoic acid), amino acid (Lys, lysine; Met, methionine; His, histidine), and mineral (P, phosphorus; Ca, calcium) compositional results and phenotypic (HSI, hepatosomatic index; VSI, viscerosomatic index; K, condition factor) measurement data amongst all fish. The coloured scale shows degree of correlation ranging from *r* = −1.0 (red) to *r* = 1.0 (blue). The size of the coloured circle indicates its significance level and circles containing asterisks are significant at *p* < 0.05. **(B)** Principal component analysis (PCA) of the same multivariate dataset with distinction between rearing temperature treatments based on their phenotypic characteristics and transcript expression. Variable eigenvectors are coloured based on their contribution to Dimension (Dim) 1 and 2 of the PCA plot.

Figure 8A shows the degree of correlation among the expression profiles of all nine target transcripts involved in RT-qPCR validation along with fillet composition [i.e., docosahexaenoic acid (DHA), eicosapentaenoic acid (EPA), total omega-3 fatty acids (Σ ω3), lysine, methionine, histidine, phosphorus, calcium concentrations], and morphometric [i.e., Fulton’s condition factor (K), viscerosomatic index (VSI), hepatosomatic index (HSI)] measurements of all fish from this study. While we detected numerous significant relationships between variables, some are particularly noteworthy. For example, DHA concentrations in the fillet were negatively correlated with expression of *thrsp*, *hacd2*, and *fabpi*. Numerous transcripts (i.e., *elovl2*, *thrsp*, *s27a2*, *hacd2*, *hgfa*, *fabpi*) also exhibited a negative relationship to lysine and/or methionine fillet concentrations. In contrast, *hsp90ab1* showed a positive correlation with both lysine and methionine concentrations. The PCA seen in Figure 8B illustrates that each temperature group clustered separately in multivariate space, with the 13.5°C aggregated between the other two treatments. The eigenvectors of the target transcripts influenced Dimension 1 (Dim1) the most (i.e., the top five contributing variables to Dim1 were *hacd2*, *thrsp*, *hgfa*, *fabpi*, and *s27a2*), while phenotypic data contributed more so to Dimension 2 (Dim2) (i.e., the top five contributing variables to Dim2 were EPA, Σ ω3, DHA, HSI, and P). Altogether, this information helped elucidate which factors are most relevant in helping differentiate temperature treatments in this study and allowed for transcriptional responses to be related to phenotypic measurements.

## Discussion

The aim of this study was to compare the transcriptomic responses in the liver of AAS reared at either 10.5, 13.5 or 16.5°C using RNA-seq. Comparing results from DESeq2 and edgeR differential expression analyses, the greatest number of DETs was found when comparing the 10.5 and 16.5°C temperature groups. That comparison showed that temperature elicits broad effects on numerous biological systems in the livers of AAS, many related to metabolism, as evidenced by finding 282 enriched GO terms. Pathway analysis further illustrated that at elevated temperature, catabolism-, stress- and apoptosis-related transcripts were upregulated in comparison to 10.5°C. In contrast, expression of oxidoreductase activity- and lipid/fatty acid metabolism-relevant transcripts was downregulated at 16.5°C. Our results were validated by qPCR, with eight out of nine metabolic-related transcripts tested positively correlated to the RNA-seq data.

It was important to size-match salmon from the separate rearing temperatures for this study, as previous findings have shown that life stage significantly impacts hepatic transcriptomic responses in diploid and triploid Atlantic salmon (Odei et al., 2020). Also, as evidenced by the results of Ignatz et al. (2020b), several phenotypic differences were observed when comparing AAS reared at these three temperatures as they grew from first feeding fry up to 1,500 g. The fact that these fish were all reproductively sterile, monosex and genetically related also limited variation, allowing for a better assessment of temperature-mediated effects.

A stringent approach was taken when assessing DETs, whereby only transcripts found differentially expressed by both DESeq2 and edgeR were included in downstream GTEA and pathway analyses. In this study, more DETs were found by DESeq2 compared to edgeR, a general trend that has been previously reported (Polinski et al., 2016; Hall et al., 2021). In Tomalty et al. (2015), juvenile diploid Chinook salmon (*Oncorhynchus tshawytscha*) were held in 12°C and then exposed to higher temperatures (i.e., 15, 18, 21, or 25°C) for 3 h followed by a 1 h recovery at 12°C. These authors reported a smaller number of DETs when comparing 12 vs. 15°C (9 DETs) or even 12 vs. 18°C (120 DETs) in gill tissue. These data differ from the results presented herein and could be explained by the difference in tissue sampled (i.e., gill vs. liver), the difference in study species, and/or the possibility that chronic exposure (i.e., after 10–12 months) to a different temperature may lead to more extensive changes in the transcriptome compared to acute thermal challenges. The latter point is further supported, wherein only 243 and 88 DE genes were identified in the liver of juvenile Atlantic salmon exposed to 23°C for 6 and 24 h, respectively compared to fish held at 13°C (Shi et al., 2019). This shows that exposure to even more extreme temperatures for short durations does not impact the transcriptome as much as shown in the current study between 10.5 and 16.5°C. However, this result could also potentially be influenced by life stage of the fish.

In 2.5 kg Atlantic salmon reared in saltwater, triploids exhibited a greater basal cortisol response when exposed to gradual increases in temperature up to 18°C compared to diploids (Sambraus et al., 2018). Further, cortisol levels increased at 18°C as compared to lower rearing temperatures (i.e., 9–15°C) in triploids (Sambraus et al., 2018). These results reflect that at 18°C, triploid Atlantic salmon experience chronic stress, a feature in agreement with the current study with AAS reared at 16.5°C as reflected in the GO term enrichment and pathway analyses. It is noteworthy that differences in basal cortisol concentrations of AAS were not detected between 10.5, 13.5, and 16.5°C at 800 g previously (Ignatz et al., 2020a). However, several classic indicators of heat stress were either upregulated [e.g., *hsp90ab1*, *serpinh1* (alias *hsp47*, encoding Serpin H1)] or downregulated [e.g., mitochondrial uncoupling protein 2 (*ucp2*), cold-inducible RNA-binding protein (*cirbp*), peroxiredoxin 6 (*prdx6*)] at 16.5°C compared to 10.5°C (Supplementary Table S1). These markers align with previous studies that investigated expression differences and DNA methylation dynamics in the liver between non-transgenic Atlantic salmon reared at 12 and 20°C (Beemelmanns et al., 2021a; Beemelmanns et al., 2021b; Beemelmanns et al., 2021c). Several of these genes have also been identified as differentially expressed during heat stress in other salmonids across a variety of tissues (Jeffries et al., 2012; Jeffries et al., 2014; Tomalty et al., 2015; Tan et al., 2016; Shi et al., 2019; Quan et al., 2021). Further study is recommended to parse out potential separate effects of triploidy and growth hormone transgenesis on thermally responsive transcripts of Atlantic salmon.

In both juvenile Atlantic salmon and brook charr (*Salvelinus fontinalis*), triploids had lower erythrocyte levels of several heat shock proteins, including HSP90, compared to diploids at several acclimation temperatures (i.e., 5, 15, and 25°C) (Saranyan et al., 2017). Lower basal levels of these proteins were hypothesized to limit triploid salmonids in their ability to prevent protein damage or denaturation at high temperatures (Saranyan et al., 2017). In the current study, expression of *hsp90ab1* was upregulated at 16.5 and 13.5°C compared to 10.5°C. It may be that even at 13.5°C, AAS are attempting to compensate for their lower basal protein synthesis by upregulating expression of *hsp90ab1* to prevent protein degradation, but further study is warranted. Similarly, with significantly higher expression of *serpinh1* at 16.5 vs. 10.5°C, it has been suggested that Atlantic salmon attempt to synthesize and stabilize collagen molecules to maintain hepatocyte structures at high temperatures (Beemelmanns et al., 2021b; Beemelmanns et al., 2021c). Additionally, Serpin H1 is thought to help neutralize reactive oxygen species (ROS) during heat stress as evidenced by increased transcript expression observed in rainbow trout (*Oncorhynchus mykiss*) (Wang et al., 2016). Thus, by upregulating *serpinh1* at 16.5°C, AAS are likely attempting to reestablish cellular homeostasis by eliminating ROS.

In contrast, downregulating *ucp2* at 16.5°C might help reduce mitochondrial ROS formation by increasing mitochondrial coupling (Laskowski et al., 2016). In fact, studies have shown that long-term acclimation to 20°C in diploid Atlantic salmon significantly reduces mitochondrial ROS production when exposed to higher temperatures compared to salmon acclimated to 12°C (Gerber et al., 2021; Gerber et al., 2020). The lower expression of *cirbp* at 16.5°C compared to 10.5°C is also indicative of heat stress, as this transcript is routinely downregulated at elevated temperature but upregulated at lower temperatures in salmonids and other vertebrates (Zhong and Huang, 2017; Akbarzadeh et al., 2018; Beemelmanns et al., 2021b; Beemelmanns et al., 2021c). This chaperone-encoding mRNA is involved in cell proliferation, survival and apoptosis and is also hypoxia sensitive (Zhong and Huang, 2017). Further, *prdx6*, which is involved in phospholipid homeostasis and lipid peroxidation repair (Fisher, 2011; Arevalo and Vázquez-Medina, 2018), was also a key downregulated transcript at 16.5°C. It has also been noted that in the liver of large (1.5–2.0 kg) immature Atlantic salmon held at 19°C for 45 days, that transcripts associated with protection against oxidative stress become downregulated compared to salmon held at 13°C (Olsvik et al., 2013). This feature is also found comparing the 10.5 and 16.5°C groups in the current study. Altogether, this suggests that AAS reared at this higher temperature may struggle with regulating oxidative stress responses.

As female triploids reduce investment in sexual maturation, this prevents the reallocation of nutrients from muscle tissue toward gonad development (Manor et al., 2014). Thus, triploid Atlantic salmon have different fillet lipid/fatty acid profiles compared to diploids (Murray et al., 2018; Everson et al., 2021). Previous findings from this experiment have also shown that rearing temperature influenced deposition rates and retention efficiencies of ω3 fatty acids (Ignatz et al., 2020b). While expression of transcripts related to fatty acid metabolism (i.e., *hacd2*, *fabpi*, *thrsp*) were negatively correlated with ω3 fatty acid composition (significant with DHA composition comparisons) within the fillet of these salmon, this likely reflects the ongoing shift in nutrient utilization of fish at 16.5°C. Although fillet ω3 fatty acids did not differ on a compositional level between rearing temperature groups at 800 g, deposition rates of total ω3, DHA, and EPA were found significantly lower during the 800–1,500 g growth period in fish reared at 16.5°C compared to the two lower temperature treatments (Ignatz et al., 2020b). Therefore, the changes in gene expression found in the current study (i.e., downregulation of fatty acid metabolism genes at 16.5°C compared to 10.5 and 13.5°C) were likely indicative of the start of this change in nutrient allocation. Ultimately, this can lead to adverse effects on final product composition and quality, as lower production and deposition of ω3 fatty acids in particular would not be ideal for the marketability of AAS. Thus, this study helps to define the optimal rearing temperature of AAS to avoid these adverse effects.

Both GTEA and pathway analysis highlighted that lipid/fatty acid metabolism was negatively impacted at 16.5°C. Similar findings have been reported in other transcriptomic studies at elevated temperature in diploid salmonids (Jeffries et al., 2012; Tomalty et al., 2015; Shi et al., 2019; Bowen et al., 2020; Beemelmanns et al., 2021c). Changes in lipid metabolism are relevant as regulation of cholesterol and phospholipids help maintain cell membrane rigidity during changes in temperature; as temperature increases, membrane structure becomes more fluid which can ultimately lead to cell death (Hazel, 1979; Fodor et al., 1995; Crockett, 1998; Farkas et al., 2001; Liu et al., 2019). “Cholesterol metabolic process” was an enriched GO term between the 10.5 and 16.5°C temperature groups, which further supports this notion. Similarly, with the downregulation of transcripts associated with the GO term “oxidoreductase activity”, this potentially offers AAS at 16.5°C less protection against oxidative stress. Several studies in diploid salmonids have shown similar results either following a heat shock or after chronic exposure to elevated temperatures (Olsvik et al., 2013; Nakano et al., 2014; Beemelmanns et al., 2021b; Beemelmanns et al., 2021c). Additionally, as catabolism, stress and apoptosis were all identified as general themes of upregulated pathways at 16.5°C, this suggests that it is not ideal for AAS to be reared at this high of a temperature.

Potentially due to differences in cellular dimensions and decreased cellular surface-to-volume ratio (Piferrer et al., 2009), triploids are also more prone to skeletal deformities and the development of cataracts (Sambraus et al., 2017b; Fraser et al., 2020; Jagiełło et al., 2021), which has led to the recommendation to increase dietary phosphorus and histidine for triploids to address each of those respective issues (Burke et al., 2010; Fjelldal et al., 2015; Taylor et al., 2015; Smedley et al., 2016). Therefore, these nutrients were important considerations in both past (Ignatz et al., 2020b) and present studies. In the current study, no correlations were found between phosphorus and histidine concentrations in the fillet with any of the target transcripts that were qPCR-validated. However, “phosphorus metabolic process” was a significantly enriched GO term that was found between the 10.5 and 16.5°C groups. Additionally, levels of methionine and/or lysine in the fillet were negatively correlated with 6 out of 7 fatty acid metabolism relevant transcripts. A previous recommendation was made that AAS may require additional methionine at temperatures >10.5°C (Ignatz et al., 2020b). It may be possible that especially at 16.5°C, the dietary requirements of AAS were not being fully met, which may have negatively impacted their metabolism of numerous nutrients.

No differences in immune responses between triploid and diploid salmonids during both bacterial and parasitic infections have been reported (Weber et al., 2013; Frenzl et al., 2014; Chalmers et al., 2016, Chalmers et al., 2017). In contrast, some evidence suggests that triploid Atlantic salmon may mount a more effective immune response against viral agents than diploids (Herath et al., 2017; Moore et al., 2017; Brown et al., 2021). In a subset of fish also assessed at 800 g from the same overarching experiment as the current study, the innate antiviral immune response was found to be significantly impacted by rearing temperature (Ignatz et al., 2020a). Significantly higher fold-change values of several immune biomarkers (i.e., *rsad2*, *isg15a*, *ifng*) were found in AAS reared at 10.5°C vs. 16.5°C comparing fish at 24 h post-injection with either PBS or pIC (Ignatz et al., 2020a). It was suggested that salmon reared at 10.5°C ultimately had a more robust innate antiviral response than both other temperature treatments (Ignatz et al., 2020a). From the current RNA-seq results, several GO terms including “immune system process”, “immune effector process”, and “activation of the innate immune response” were enriched when comparing DETs between the 10.5 and 16.5°C groups. This illustrates that temperature significantly influenced basal immune-relevant transcript expression in AAS liver, with higher expression levels at 10.5 than 16.5°C. This could mean that AAS reared at 10.5°C would be more prepared to respond to pathogens and mount a more effective immune response than if they are reared at elevated temperature as previously suggested (Ignatz et al., 2020a), but this requires further testing. While temperature did not influence basal expression of immune-relevant genes in Ignatz et al. (2020a), these results differ from what has been shown in basal expression in liver and head kidney of post-smolt diploid Atlantic salmon reared at 12 and 20°C where expression was generally found higher at elevated temperature (Zanuzzo et al., 2020; Beemelmanns et al., 2021b).

## Conclusion

Overall, the results from this study indicate that it is not optimal to rear AAS at 16.5°C due to increases in expression of transcripts related to stress, apoptosis and catabolism. Moreover, negative regulation of lipid/fatty acid metabolism is not ideal especially as it has previously been noted that deposition rates of ω3 fatty acids were lower at 16.5°C compared to 10.5 and 13.5°C (Ignatz et al., 2020b). This is the first RNA-seq study to investigate the impact of rearing temperature on the hepatic transcriptome of AAS and helps inform AquaBounty’s husbandry practices using their land-based freshwater RAS. This is important as commercialization of AquAdvantage Salmon is ongoing in the United States and Canada, and these results can be used to optimize the physiological performance of these fish. The current results are also informative as AAS appear to have responded similarly to conventional salmonids when exposed to elevated temperatures. Future work could consider optimizing the diet for AAS reared at 10.5 and/or 13.5°C and using transcriptomics to help determine best practices for their commercial production.

## Data Availability

The datasets presented in this study can be found in online repositories. The names of the repository/repositories and accession number(s) can be found in Table 1.
