# Supplementary material for: RNA-Seq Analysis of the Growth Hormone Transgenic Female Triploid Atlantic Salmon (Salmo salar) Hepatic Transcriptome Reveals Broad Temperature-Mediated Effects on Metabolism and Other Biological Processes
Source: Front Genet. 2022 May 23;13:852165. doi: 10.3389/fgene.2022.852165 (PMC9168996; doi:10.3389/fgene.2022.852165)
Supplement: Supplementary file 3 [file Image2.pdf]

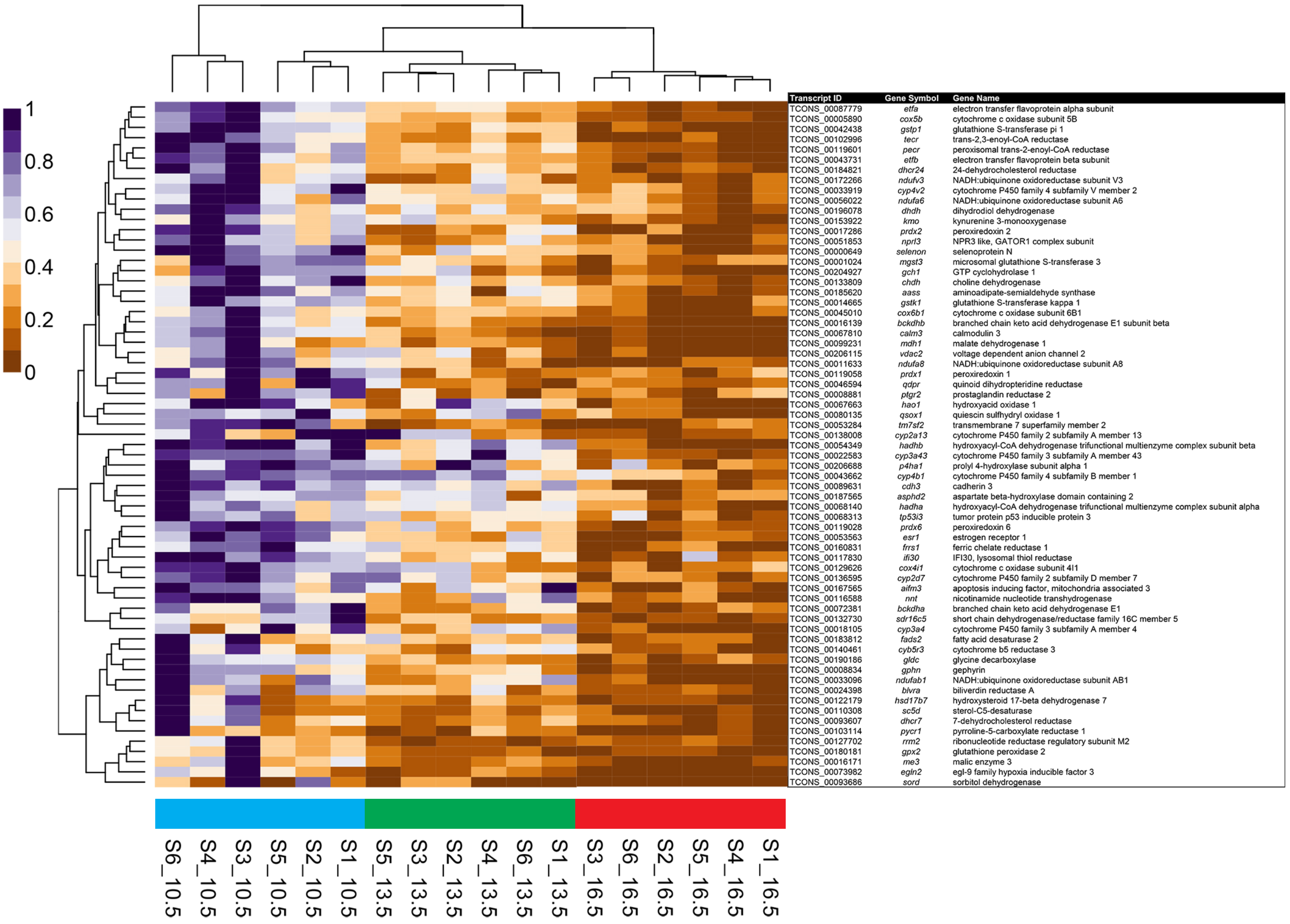

Transcript ID	Gene Symbol	Gene Name
TCONS_00087779	*etfa*	electron transfer flavoprotein alpha subunit
TCONS_00005890	*cox5b*	cytochrome c oxidase subunit 5B
TCONS_00042438	*gstp1*	glutathione S-transferase pi 1
TCONS_00102996	*tecr*	trans-2,3-enoyl-CoA reductase
TCONS_00119601	*pecr*	peroxisomal trans-2-enoyl-CoA reductase
TCONS_00043731	*etfb*	electron transfer flavoprotein beta subunit
TCONS_00184821	*dhcr24*	24-dehydrocholesterol reductase
TCONS_00172266	*ndufv3*	NADH:ubiquinone oxidoreductase subunit V3
TCONS_00033919	*cyp4v2*	cytochrome P450 family 4 subfamily V member 2
TCONS_00056022	*ndufa6*	NADH:ubiquinone oxidoreductase subunit A6
TCONS_00196078	*dhdh*	dihydrodiol dehydrogenase
TCONS_00153922	*kmo*	kynurenine 3-monooxygenase
TCONS_00017286	*prdx2*	peroxiredoxin 2
TCONS_00051853	*nprl3*	NPR3 like, GATOR1 complex subunit
TCONS_00000649	*selenon*	selenoprotein N
TCONS_00001024	*mgst3*	microsomal glutathione S-transferase 3
TCONS_00204927	*gch1*	GTP cyclohydrolase 1
TCONS_00133809	*chdh*	choline dehydrogenase
TCONS_00185620	*aass*	aminoadipate-semialdehyde synthase
TCONS_00014665	*gstk1*	glutathione S-transferase kappa 1
TCONS_00045010	*cox6b1*	cytochrome c oxidase subunit 6B1
TCONS_00016139	*bckdhb*	branched chain keto acid dehydrogenase E1 subunit beta
TCONS_00067810	*calm3*	calmodulin 3
TCONS_00099231	*mdh1*	malate dehydrogenase 1
TCONS_00206115	*vdac2*	voltage dependent anion channel 2
TCONS_00011633	*ndufa8*	NADH:ubiquinone oxidoreductase subunit A8
TCONS_00119058	*prdx1*	peroxiredoxin 1
TCONS_00046594	*qdpr*	quinoid dihydropteridine reductase
TCONS_00008881	*ptgr2*	prostaglandin reductase 2
TCONS_00067663	*hao1*	hydroxyacid oxidase 1
TCONS_00080135	*qsox1*	quiescin sulfhydryl oxidase 1
TCONS_00053284	*tm7sf2*	transmembrane 7 superfamily member 2
TCONS_00138008	*cyp2a13*	cytochrome P450 family 2 subfamily A member 13
TCONS_00054349	*hadhb*	hydroxyacyl-CoA dehydrogenase trifunctional multienzyme complex subunit beta
TCONS_00022583	*cyp3a43*	cytochrome P450 family 3 subfamily A member 43
TCONS_00206688	*p4ha1*	prolyl 4-hydroxylase subunit alpha 1
TCONS_00043662	*cyp4b1*	cytochrome P450 family 4 subfamily B member 1
TCONS_00089631	*cdh3*	cadherin 3
TCONS_00187565	*asphd2*	aspartate beta-hydroxylase domain containing 2
TCONS_00068140	*hadha*	hydroxyacyl-CoA dehydrogenase trifunctional multienzyme complex subunit alpha
TCONS_00068313	*tp53i3*	tumor protein p53 inducible protein 3
TCONS_00119028	*prdx6*	peroxiredoxin 6
TCONS_00053563	*esr1*	estrogen receptor 1
TCONS_00160831	*frrs1*	ferric chelate reductase 1
TCONS_00117830	*ifi30*	IFI30, lysosomal thiol reductase
TCONS_00129626	*cox4i1*	cytochrome c oxidase subunit 4I1
TCONS_00136595	*cyp2d7*	cytochrome P450 family 2 subfamily D member 7
TCONS_00167565	*aifm3*	apoptosis inducing factor, mitochondria associated 3
TCONS_00116588	*nnt*	nicotinamide nucleotide transhydrogenase
TCONS_00072381	*bckdha*	branched chain keto acid dehydrogenase E1
TCONS_00132730	*sdr16c5*	short chain dehydrogenase/reductase family 16C member 5
TCONS_00018105	*cyp3a4*	cytochrome P450 family 3 subfamily A member 4
TCONS_00183812	*fads2*	fatty acid desaturase 2
TCONS_00140461	*cyb5r3*	cytochrome b5 reductase 3
TCONS_00190186	*gldc*	glycine decarboxylase
TCONS_00008834	*gphn*	gephyrin
TCONS_00033096	*ndufab1*	NADH:ubiquinone oxidoreductase subunit AB1
TCONS_00024398	*blvra*	biliverdin reductase A
TCONS_00122179	*hsd17b7*	hydroxysteroid 17-beta dehydrogenase 7
TCONS_00110308	*sc5d*	sterol-C5-desaturase
TCONS_00093607	*dhcr7*	7-dehydrocholesterol reductase
TCONS_00103114	*pycr1*	pyrroline-5-carboxylate reductase 1
TCONS_00127702	*rrm2*	ribonucleotide reductase regulatory subunit M2
TCONS_00180181	*gpx2*	glutathione peroxidase 2
TCONS_00016171	*me3*	malic enzyme 3
TCONS_00073982	*egln2*	egl-9 family hypoxia inducible factor 3
TCONS_00093686	*sord*	sorbitol dehydrogenase

Supplementary Figure S2. Heatmap and hierarchal clustering of non-redundant differentially expressed transcripts (67 DETs) found in comparison of the 10.5 °C and 16.5 °C groups associated with GO:0016491 - Oxidoreductase Activity. RNA samples used in sequencing are labeled on the base of the figure, with standardized fragments per kilobase of transcript per million mapped reads (FPKM) values for each DET clustered along the vertical axis.
