# Supplementary material for: RNA-Seq Analysis of the Growth Hormone Transgenic Female Triploid Atlantic Salmon (Salmo salar) Hepatic Transcriptome Reveals Broad Temperature-Mediated Effects on Metabolism and Other Biological Processes
Source: Front Genet. 2022 May 23;13:852165. doi: 10.3389/fgene.2022.852165 (PMC9168996; doi:10.3389/fgene.2022.852165)
Supplement: Supplementary file 8 [file Image1.pdf]

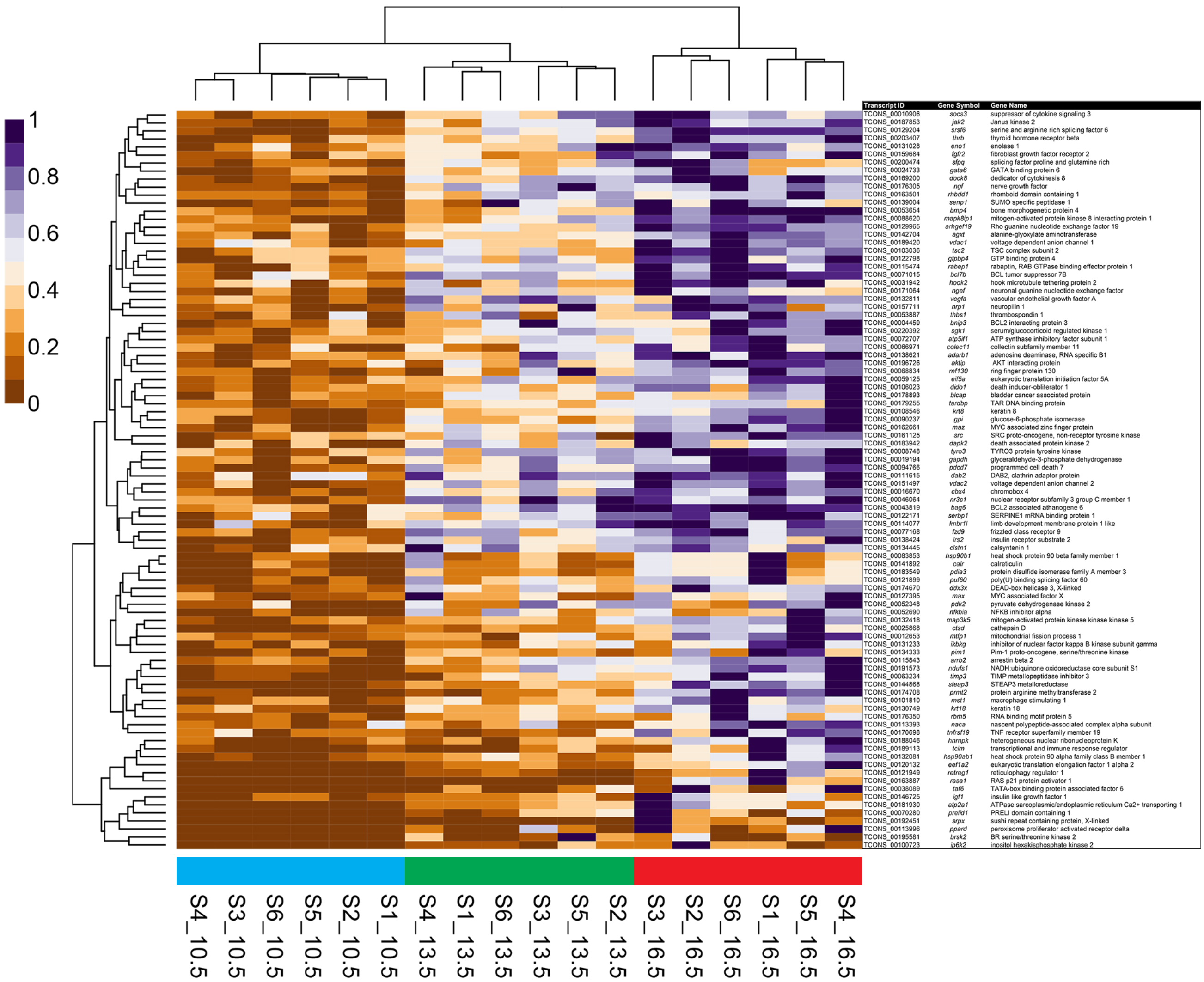

Supplementary Figure S1. Heatmap and hierarchal clustering of non-redundant differentially expressed transcripts (92 DETs) found in comparison of the 10.5 °C and 16.5 °C groups associated with GO:0006915 - Apoptotic Process. RNA samples used in sequencing are labeled on the base of the figure, with standardized fragments per kilobase of transcript per million mapped reads (FPKM) values for each DET clustered along the vertical axis.
